# Supplementary material for: A Pilot Study of Exosome Proteomic Profiling Reveals Dysregulated Metabolic Pathways in Endometrial Cancer
Source: Biomedicines. 2025 Jan 3;13(1):95. doi: 10.3390/biomedicines13010095 (PMC11759861; doi:10.3390/biomedicines13010095)
Supplement: Supplementary file 1 [file biomedicines-13-00095-s001.zip › Figure S3.pdf]

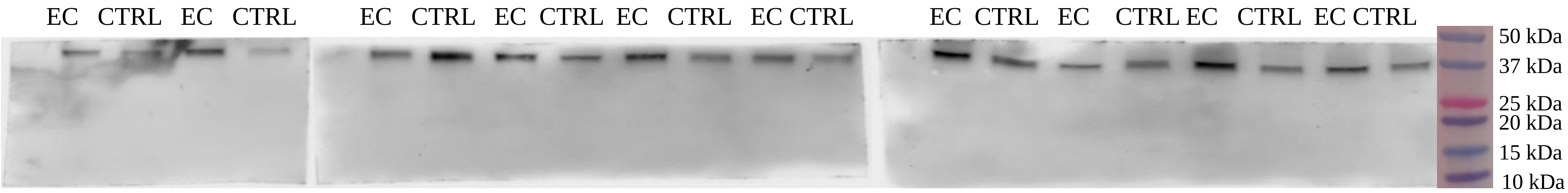

The whole membrane of protein PRDX2

CTRL-control
EC- endometrial cancer

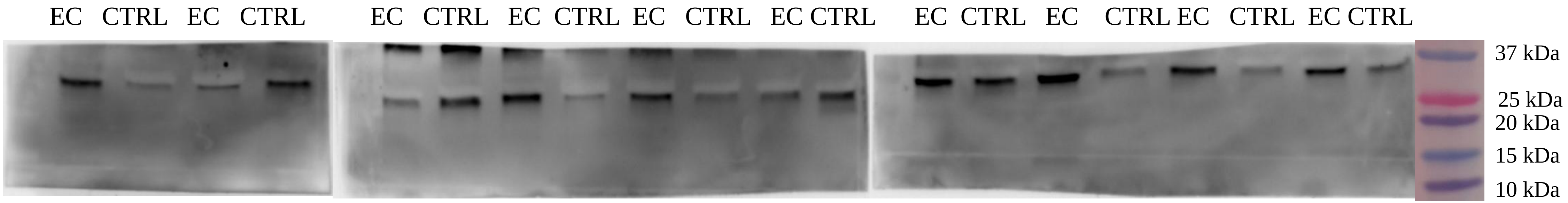

The whole membrane of protein GADPH

CTRL-control
EC- endometrial cancer

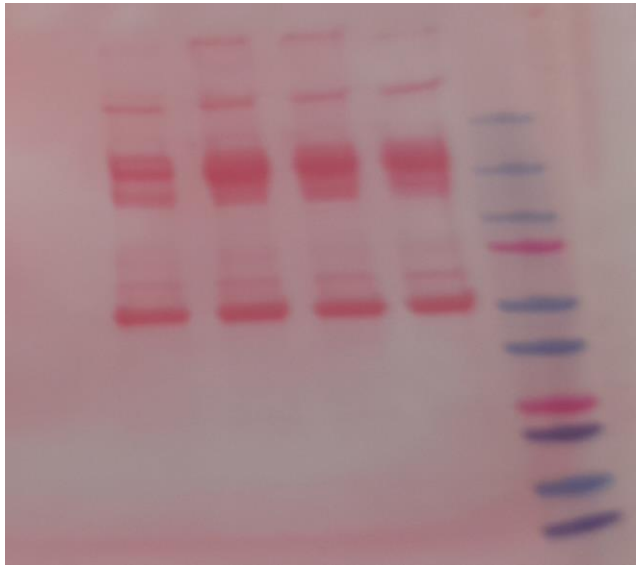

Serum exosome membrane 3

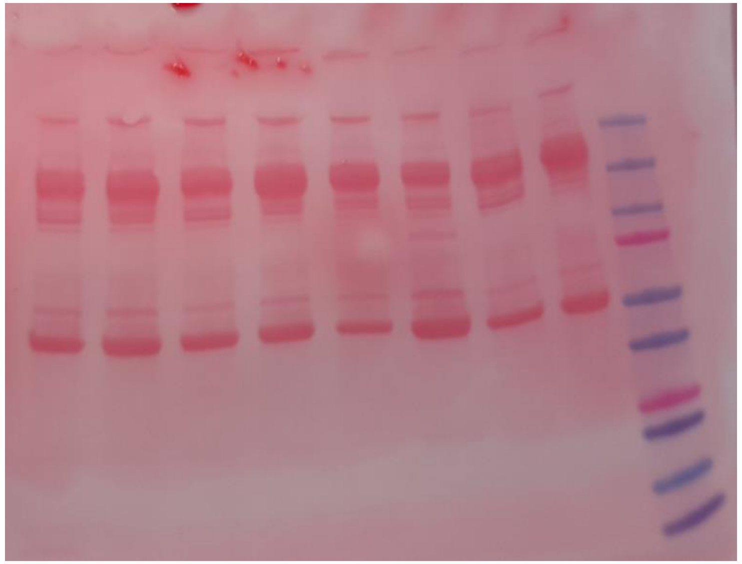

Serum exosome membrane 2

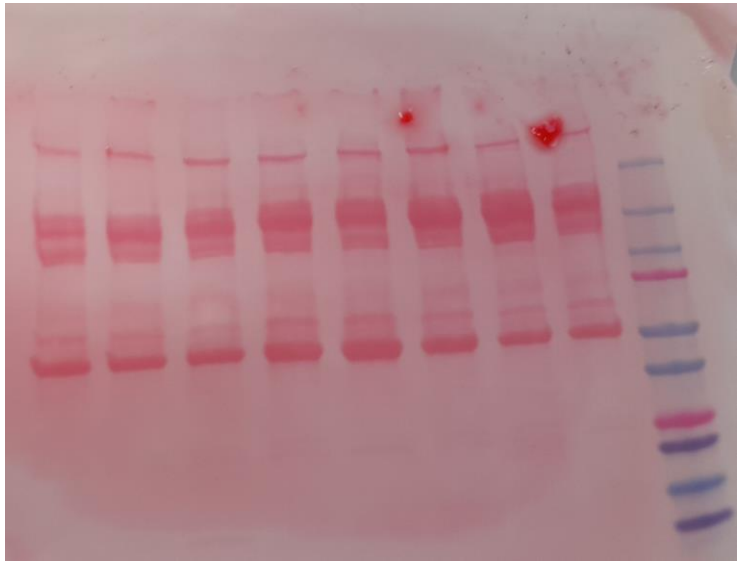

Serum exosome membrane 1
