# Supplementary material for: A Pilot Study of Exosome Proteomic Profiling Reveals Dysregulated Metabolic Pathways in Endometrial Cancer
Source: Biomedicines. 2025 Jan 3;13(1):95. doi: 10.3390/biomedicines13010095 (PMC11759861; doi:10.3390/biomedicines13010095)
Supplement: Supplementary file 1 [file biomedicines-13-00095-s001.zip › Table S1.pdf]

Supplementary Table S1: Clinico-pathological characteristics of the 20 EC women enrolled in the study.

Sample number	Age	Histotype (Grade)	Tumor dimensions (cm)	TNM Classification of Malignant Tumours, 7 Edition	Stage	Methodology
176	56	Endometroid adenocarcinoma (G1)	4,6	pT1a G1 LV- R0	I A	LC-MS/MS
140	75	Endometroid adenocarcinoma (G3)	Macroscopically not evaluate	pT1a N0 G3 LVI+ R0	IA	LC-MS/MS
157	71	Endometroid adenocarcinoma (G2)	3,4	pT1a N0(sn)(i-) (0/4)	I A	LC-MS/MS
172	80	Endometroid adenocarcinoma (G2)	4x3	pT1b N0 (sn)(i-) G2 LVI + R0	IB	LC-MS/MS
199	61	Endometroid adenocarcinoma (G1)	22 x 15 e 24 x 18	pT1a N0(sn)(i-) G1 LVI - R0	IA	LC-MS/MS
179	84	Endometroid adenocarcinoma (G1)	1.2	pT1b N0 (sn) (i-) G1 LVI- R0	IB	LC-MS/MS
142	71	Endometroid adenocarcinoma (G3)	3.4x2.6x1	pT1a N0 (sn)(i-) G3 LVI+ R0	I A	LC-MS/MS
190	57	Endometroid Adenocarcinoma (G1)	3 x 2.5 x 0.5	pT1a N0(sn)(i-) G1 LVI- R0	IA	LC-MS/MS
160	76	Endometroid adenocarcinoma (G2)	3x2.5x2	pT1b N0(sn)(i-) V0 R0	IB	LC-MS/MS
165	62	Endometroid adenocarcinoma (G2)	2x1.5	pT1a G2 N0	IA	LC-MS/MS
190	57	Endometroid adenocarcinoma G1	3 x 2.5 x 0.5	pT1a N0(sn)(i-) G1 LVI- R0	IA	Western-blotting
135	58	Endometroid adenocarcinoma (G3)	Macroscopically not evaluate	pT1b N0 G3 LV1 R0	IB	Western-blotting

273	78	Endometroid adenocarcinoma G1	1.2	pT1a N0(sn)(i-) G1 LVI- R0	IB	Western-blotting
164	65	Endometroid adenocarcinoma G1	Macroscopically not evaluate	pT1b N0 (sn)(i-) G1 LVI - R0	IB	Western-blotting
223	58	Endometroid adenocarcinoma G1	4.7x2.2	pT1a N0 (sn)(i-) G1 LVI-R0	IA	Western-blotting
272	76	Endometroid adenocarcinoma G1	5.5x2.5x1.5	pT1a G1 LVI+ (focal) R0. MMRd.	IA	Western-blotting
271	76	Endometroid adenocarcinoma G2	6,5x4,5	pT1b N0 (sn)(i-) G2 LVI+ (focal) R0 . MMRp	IB	Western-blotting
168	74	Endometroid adenocarcinoma G3	macroscopicamente non valutabile	pT1a N0 G3 LVI - R0	IA	Western-blotting
163	81	Endometroid adenocarcinoma G2	macroscopicamente non valutabile	pT2 G2 LVI + R0	IA	Western-blotting
209	42	adenocarcinoma endometrioide G1	2,5x1,9x0,5	pT1a N0 (sn)(i-) V0 Pn0 R0	IA	Western-blotting
