# Supplementary figures and images for: A Pilot Study of Exosome Proteomic Profiling Reveals Dysregulated Metabolic Pathways in Endometrial Cancer
Source: Biomedicines. 2025 Jan 3;13(1):95. doi: 10.3390/biomedicines13010095 (PMC11759861; doi:10.3390/biomedicines13010095)

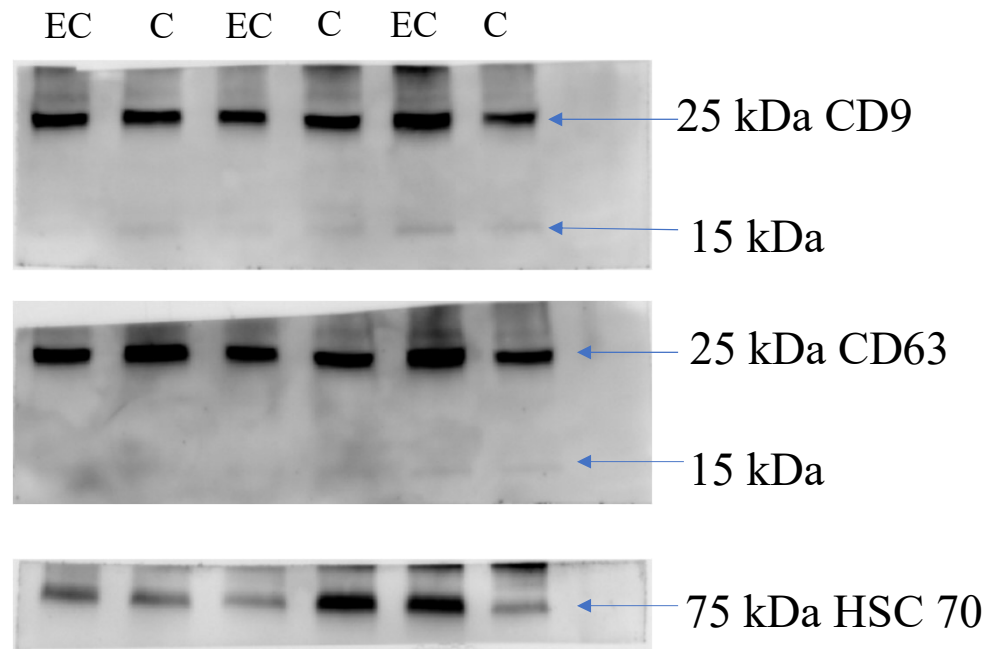

C-control  
EC- endometrial cancer

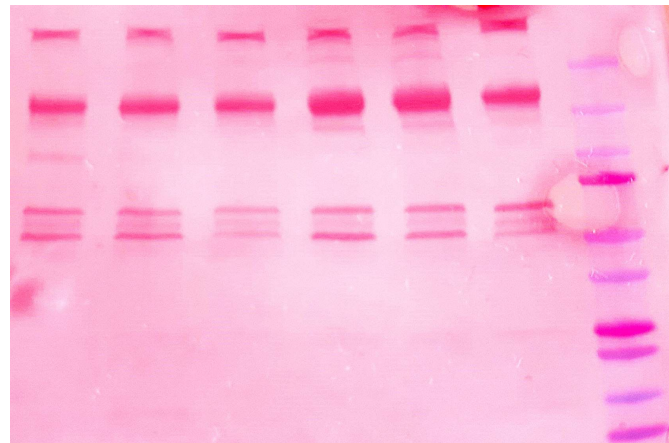

Whole membrane stained with red ponceau

Supplement: Supplementary file 1 [file biomedicines-13-00095-s001.zip › Figure S1.pdf]
